# Determination of the relationship between satisfaction with prenatal care and satisfaction with and perception of childbirth

**DOI:** 10.1590/1806-9282.20251085

**Published:** 2026-05-01

**Authors:** Rukiye Demir, Toumpa Boulmpoul

**Affiliations:** 1 Çanakkale Onsekiz Mart University, Faculty of Health Sciences, Department of Midwifery – Çanakkale, Türkiye.

**Keywords:** Mother, Pregnancy, Prenatal care

## Abstract

**OBJECTIVE:**

The aim of this study was to determine the relationship between satisfaction with prenatal care and labor satisfaction and perception of labor.

**METHODS:**

The cross-sectional and correlational study was conducted in the obstetrics ward of a public hospital. The population of the study consisted of women who gave birth in this hospital, while the sample consisted of 200 women. The data were collected face-to- face using the "Personal Information Form, Prenatal Care Satisfaction Scale, Birth Satisfaction Scale, and Mother's Perception of Childbirth Scale."

**RESULTS:**

It was found that 56% of the mothers who participated in the study received prenatal care from a midwife/nurse, 60.3% received prenatal care at a Family Health Center, and the mean total scores of the mothers were 71.69±9.67, 73.66±5.48, and 102.56±5.26 on the Prenatal Care Satisfaction Scale, Mother's Perception of Childbirth Scale, and Birth Satisfaction Scale, respectively. In addition, a positive and significant correlation was found between the mean scores of the women on the Prenatal Care Satisfaction Scale and the mean scores on the Mother's Perception of Childbirth Scale and Birth Satisfaction Scale.

**CONCLUSION:**

In the study, it was concluded that as women's prenatal care satisfaction levels increased, their birth satisfaction and positive birth perception also increased.

## INTRODUCTION

Birth satisfaction, which is accepted as an indicator of quality in care, is defined as "meeting women's expectations and wishes at birth" and birth perception is defined as "women's experiences at birth and the meaning they attribute to these experiences."^1–5^ It is extremely important for a woman to have a high level of perception and satisfaction during pregnancy and labor^6–8^. Studies have reported that 27–75% of women perceive the birth process as a negative experience, with prenatal care affecting birth satisfaction and perception in various ways^9,10^. For this reason, negative birth experience and perception has been frequently addressed in birth studies conducted in recent years, especially in developed countries^7,11^. In addition, available evidence shows that providing women with high satisfaction and quality prenatal care positively affects maternal and fetal health and improves birth outcomes, has a significant effect on the reduction of poor birth outcomes, increases birth satisfaction and positive birth perception, provides coping and control of fear of childbirth, and reduces maternal morbidity and mortality rates, and the risk of stillbirth and preterm birth^12–16^. Low prenatal care satisfaction may lead to negative conditions such as postpartum depression, posttraumatic stress disorder, desire for cesarean section in subsequent deliveries, sexual dysfunction, inadequacy in mother-infant attachment, and breastfeeding problems^8,17^. For this reason, the World Health Organization (WHO) emphasizes the necessity of determining satisfaction with prenatal care, assessing unmet needs and planning interventions for these needs, increasing women's satisfaction with childbirth, and developing a positive perception of childbirth. In addition, WHO recommends evaluating and increasing the quality of prenatal care to achieve a positive perception of birth and enhance birth satisfaction^18^. The aim of this study is to examine the relationship between women's satisfaction with prenatal care and their satisfaction with and perception of childbirth.

## METHODS

The cross-sectional and correlational study was conducted from November 2023 to 2024. The population of the study conducted in the obstetrics service of a state hospital included women who gave birth hospital. The number of women who applied to this hospital and gave birth within 1 year (2022) was 1,228. Accordingly, the study population was calculated using the known sampling formula [n=(N.t2.p.q)/(d2). (N-1)+(t2.p.q)], with a 95%CI and a 5% margin of error. The sample was found to be 164, and the study was completed with 200 people to account for possible data losses (n=200).

### Data collection

The data of the study were collected with the "Personal Information Form, Prenatal Care Satisfaction Scale (PCSS), Birth Satisfaction Scale (BSS), and Mother's Perception of Childbirth Scale (MPCS)."

Personal information: The Personal Information Form consisted of 15 questions in total: questions prepared by reviewing the literature^6,11,12,19,20^.

PCSS was developed to measure satisfaction with prenatal care and was adapted into Turkish by Aslantekin. The 22-item Likert-type scale has a minimum score of 22 and a maximum score of 110^19^.

BSS: Short form was conducted in Turkey by Serhatlıoglu et al. A scale consisting of 10 items determines the birth satisfaction level of mothers. A low score of 0 and a maximum score of 40 are obtained, and the higher the score, the higher the birth satisfaction level^8^.

MPCS was its Turkish validity and reliability study was conducted by Güngör and Beji. The sub-dimensions of the 25-item, five sub-dimensional Likert-type scale^20^.

Before the application, the women who met the inclusion criteria were informed about the purpose of the study, and verbal and written consent was obtained from them. The questionnaire was completed by face-to-face interview technique, and then the women were asked to fill in the scale themselves.

### Data analysis

The IBM SPSS Statistics 24 program was used for statistical analyses. Descriptive statistics were given as a number, percentage, a mean, and a standard deviation. The Kolmogorov-Smirnov test was used to determine whether the data were normally distributed. In group comparisons, the chi-square test was used for categorical data, the independent samples t-test and the Mann-Whitney U test were used for paired groups, and the Kruskal-Wallis test was used for comparisons of more than two groups. Spearman Correlation Analysis was used to determine the relationships between the scales. The statistical significance level was accepted as p<0.05.

### Ethical considerations

Ethics committee permission (Number: E-84026528-050.01.04-2300181820 and date: 4.08.2023) was obtained from the Canakkale Onsekiz Mart University Graduate Education Institute Ethics Committee, and institutional permission (Number: E-97769597-799-229063107 and date: 10.11.2023) was obtained from the State Hospital. Permission for the use of the scale was obtained by e-mail. Written and verbal consent of the women participating in the study was obtained.

## RESULTS

The mean age of the women who participated in the study was 28.64±95.12 years, 49% were high school graduates, 62.5% were housewives, 52% had poor income perception, 71% had nuclear families, 59% were multiparous, and 54% had unplanned pregnancies. In addition, it was found that 56% of the women received prenatal care from a midwife/nurse and 60.3% received it at a Family Health Center (Table 1).

**Table 1:** Distribution of socio-demographic and obstetric characteristics of women (n=200).

Features		n	%
Age			
	18–26	86	(43.0)
	27–34	72	(36.0)
	35 years and above	42	(21.0)
Mean age±SD* 28.64±95.12			
Education status			
	Primary/secondary school	34	(17.0)
	High school	98	(49.0)
	University	68	(34.0)
Employment status			
	Working	75	(37.5)
	Not working (housewife)	125	(62.5)
Perception of income status			
	Bad	104	(52.0)
	Medium/good	96	(48.0)
Family type			
	Nuclear family	142	(71.0)
	Extended family	58	(29.0)
Parity status			
	Primiparous	82	(41.0)
	Multipar	118	(59.0)
Planned pregnancy status			
	Yes	98	(46.0)
	No	102	(54.0)
The person who received prenatal care			
	Midwife/nurse	112	(56.0)
	Doctor	88	(44.0)
Where she received prenatal care in this pregnancy**			
	Family Health Center	164	(60.3)
	State Hospital Private	52	(19.1)
	Hospital University	34	(12.5)
	Hospital	22	(8.1)

* Standard deviation,

** More than one option is marked.

SD: standard deviation.

When the mean total scores of the women who participated in the study were examined, they were 71.69±9.67, 73.66±5.48, and 102.56±5.26 on the PCSS, MPCS, and BSS, respectively (Table 2).

**Table 2:** Distribution of women's mean total scores on the Prenatal Care Satisfaction Scale, Mother's Perception of Childbirth Scale, and Birth Satisfaction Scale (n=200).

Scales		Available for purchase Min–max points	Received Min–max points	Mean±SD
PCSS	The art of care	7–35	17–29	22.38±2.91
	Technical quality	4–20	8–16	12.59±1.67
	Accessibility	4–20	9–17	11.47±3.62
	Physical environment	4–20	8–16	13.35±1.73
	Compliance	3–15	8–12	11.69±1.24
	Total	22–110	22–102	71.69±9.67
MPCS	Experiences at the moment of birth	7–35	14–18	17.82±2.46
	Experiences in the pain period of labor	7–35	16–21	20.1±1.52
	Postpartum	4–20	14–15	14.35±3.24
	Spouse participation	4–20	7–14	12.70±1.73
	Awareness	3–15	8–9	8.63±6.51
	Total	25–125	25–123	73.66±5.48
BSS	Quality of care	8–40	19–39	29.45±1.65
	Personal characteristics of women	8–40	19–35	28.46±2.47
	Experiencing stress in childbirth	14–70	22–65	44.65±2.31
	Total	30–150	61–134	102.56±5.26

PCSS: Prenatal Care Satisfaction Scale; MPCS: Mother's Perception of Childbirth Scale; BSS: Birth Satisfaction Scale.

When the relationship between the scores of the women on the PCSS and the MPCS and its sub-dimensions was examined, it was found that there was a positive and significant relationship between the total score of the PCSS and the total score of the MPCS (r: 0.315, p: 0.000). It was found that there was a positive and significant relationship between the sub-dimensions of Experiences at the Moment of Birth (r: 0.295, p: 0.000), Experiences in the Pain Period of Childbirth (r: 0.314, p: 0.001), and Awareness (r: 0.560, p: 0.026), and the total score of the PCSS. The relationship between women's PCSS and BSS total scores and its sub-dimensions was examined, and it was found that there was a positive and significant relationship between the PCSS total score and the BSS total score (r: 0.424, p: 0.001).

In addition, it was found that there was a positive and significant relationship between the Quality of Care (r: 0.272, p: 0.011) and Stress Experience in Childbirth (r: 0.225, p: 0.001), which are sub-dimensions of the BSS, and the total score of the PCSS (Table 3). In other words, women's satisfaction with prenatal care was significantly associated with increased labor satisfaction and perception of labor (p<0.05).

**Table 3:** Between the Prenatal Care Satisfaction Scale and the total and sub-dimensions of the Mother's Perception of Childbirth Scale and Birth Satisfaction Scale.

Scales	Experiences at the moment of birth	Experiences during the pain period of labor	Postpartum	Spouse involvement	Becoming aware	MPCS Total	Quality of care	Personal characteristics of women	Experiencing stress in childbirth	BSS total
The art of care	r=0.342** p=0.000	r=0.234* p=0.032	r=0.253 p=0.085	r=0.310** p=0.000	r=0.154** p=0.000	r=0.273** p=0.000	r=0.210** p=0.000	r=0.354 p=0.627	r=0.271* p=0.023	r=0.328* p=0.013
Technical quality	r=0.364 p=0.180	r=0.207* p=0.043	r=0.373 p=0.135	r=0.358 p=0.712	r=0.207 p=0.062	r=0.352 p=0.089	r=0.354* p=0.049	r=0.225 p=0.376	r=0.143 p=0.127	r=0.265 p=0.079
Accessibility	r=0.315* p=0.010	r=0.382** p=0.000	r=0.344* p=0.045	r=0.315 p=0.236	r=0.392* p=0.012	r=0.324* p=0.014	r=0.312** p=0.000	r=0.342 p=0.612	r=0.344** p=0.000	r=0.286** p=0.000
Physical environment	r=0.411** p=0.000	r=0.386** p=0.000	r=0.216** p=0.000	r=0.411 p=0.356	r=0.386** p=0.000	r=0.236* p=0.020	r=0.401* p=0.023	r=0.286 p=0.389	r=0.256** p=0.000	r=0.372* p=0.018
Compliance	r=0.256 p=0.079	r=0.462 p=0.356	r=0.253 p=0.124	r=0.256 p=0.689	r=0.542 p=0.321	r=0.253 p=0.124	r=0.256 p=0.015	r=0.562 p=0.189	r=0.256 p=0.075	r=0.345 p=0.065
PCSS Total	r=0.295** p=0.000	r=0.314* p=0.001	r=0.245 p=0.320	r =0.256 p=0.890	r=0.560* p=0.026	r=0.315** p=0.000	r=0.272* p=0.011	r=0.304 p=0.235	r=0.225* p=0.001	r=0.424* p=0.001

* <0.05,

** p<0.001,

r=Spearman correlation analysis. MPCS: Mother's Perception of Childbirth Scale; BSS: Birth Satisfaction Scale; PCSS: Prenatal Care Satisfaction Scale.

## DISCUSSION

In the study conducted to determine the relationship between prenatal care satisfaction and birth satisfaction, it was observed that more than half of the women received prenatal care from a midwife or nurse and at an antenatal service center. It is observed that different results have been obtained in similar studies conducted on the subject in the literature. It was reported that women received prenatal care mostly from physicians^1,14^. In our study, women mostly received prenatal care from midwives, possibly because they predominantly sought care in Family Health Centers (FHCs), and pregnancy follow-ups in FHCs are performance-based. Existing evidence suggests that providing women with prenatal care that is highly satisfactory has a significant impact on reducing poor birth outcomes. For this reason, studies should be conducted to ensure that pregnant women receive more prenatal care, caregivers are more willing to provide it, and both the rate and quality of care increase.

It was determined that the prenatal care satisfaction levels of the women participating in the study were satisfactory. When examined in the literature, it is seen that the results of the study align with previous studies. Similar to our study, it was determined in the studies conducted by Yağmur and Tuz Doğaner and Bal et al. that the mean prenatal care satisfaction score of pregnant women was good^14,21^. It has been reported that increasing satisfaction with prenatal care services is important for establishing and improving maternal and child health. Therefore, it is important for healthcare personnel providing prenatal care to evaluate women's satisfaction with the care provided and their opinions about it, to identify and eliminate factors that negatively affect satisfaction, to increase the quality of perinatal care, and to plan interventions to address these needs by evaluating unmet needs.

It was determined that the birth perception of the women participating in the study was at a moderate level. Similar to our study, Yılmaz and Nazik found that women's perception of childbirth was at a moderate level^2^. In another study, it was reported that 7–35% of women perceived the birth process as a negative experience^13^. Although women's perception of labor is affected by many factors, the care given in the prenatal and intrapartum period may influence it as well. For this reason, midwives should not forget that they have important responsibilities in eliminating the factors that will negatively affect births, reducing women's fears and anxieties related to birth, preparing them for labor, and ensuring that they have a positive birth experience.

It was determined that the birth satisfaction levels of the women participating in the study were good. Our research result is in line with the literature. In fact, in the study conducted by Bilgin et al., it was determined that women's satisfaction with birth was at a good level, that women who gave birth vaginally were more satisfied, and that women who stated that their birth experience was positive also had better birth satisfaction^16^. In addition, Özcan and Aslan found that women's satisfaction levels regarding childbirth in Van were low^17^. It is thought that the difference in the results of the study may be because the women participating in the study had normal vaginal deliveries, women's perceptions of childbirth were not low, or the regions where the study was conducted were different. In addition, interventions made during the birth process change the course of labor and negatively affect satisfaction with childbirth. Implementing the factors affecting childbirth satisfaction and these needs is also necessary.

The perception of prenatal care may also affect the perception of childbirth. It was determined that as the prenatal care satisfaction levels of the women in the study increased, the perception of birth also increased. Although there is no study evaluating perinatal care practices and maternal perception of birth in the literature, there are studies evaluating the effect of intrapartum care and support on the perception of birth. In a study conducted by Sarıboğa and Zeyneloğlu, it was concluded that the perception of nursing care among women who had a positive perception of birth was also positive^4^. In addition, Yılmaz and Nazik found that there was a positive and significant correlation between the mean scores of the MPCS, and all its sub-dimensions, and the mean scores of the Nursing Care Perception Scale^2^. As seen in the results of our study and other studies, the care provided in the prenatal period and satisfaction with care can prevent women from having a negative birth experience by improving their coping with childbirth.

It was determined that as the satisfaction levels of the women participating in the study increased due to the prenatal care they received, their satisfaction with childbirth also increased. When the literature was examined, Bal et al. conducted a study to determine the relationship between prenatal care satisfaction and birth satisfaction and found that, similar to our study, women's satisfaction with birth was significantly high increased as their satisfaction with prenatal care increased^14^. In addition, Mohammad et al. evaluated maternal satisfaction in Jordan and found that 75.6% of postpartum women were dissatisfied with the care services they received during delivery^22^. They also found that pregnant women who received education and information had more trust in healthcare personnel and had higher satisfaction levels. This difference may be related to the fact that our study was conducted only with vaginal deliveries and that the individuals also participated in childbirth preparation classes and were informed about the birth process. These findings are important as a guide for clinical caregivers. Furthermore, these results are considered important for future research.

## LIMITATIONS

This study includes only the statements of women in the hospital where the study was conducted. In addition, the number of mothers sampled was limited due to the low rate of normal births in the hospital where the study was conducted. Additionally, the data in this study may be biased because they were based on participants’ self-reports.

## CONCLUSION

In the study, it was concluded that women received prenatal care mostly from midwives/nurses and at FHCs, their prenatal care and birth satisfaction levels were good, and their perception of birth was moderate. Additionally, as women's prenatal care satisfaction levels increased, their perception and satisfaction with birth also increased. It is recommended that more studies be conducted to determine women's satisfaction with prenatal care and to plan interventions for these needs by evaluating the unmet needs, to search for ways to increase women's satisfaction with childbirth and to develop a positive perception of childbirth, and to use the results of the study in the planning and implementation of services in order to provide quality prenatal care services.

## Data Availability

The datasets generated and/or analyzed during the current study are available from the corresponding author upon reasonable request.
